# Spatiotemporal Dynamics of Dissemination of Non-Pandemic HIV-1 Subtype B Clades in the Caribbean Region

**DOI:** 10.1371/journal.pone.0106045

**Published:** 2014-08-22

**Authors:** Marina Cabello, Yaxelis Mendoza, Gonzalo Bello

**Affiliations:** 1 Laboratório de AIDS e Imunologia Molecular, Instituto Oswaldo Cruz, FIOCRUZ, Rio de Janeiro, Brazil; 2 Department of Genomics and Proteomics, Gorgas Memorial Institute for Health Studies, Panama City, Panama; 3 Department of Biotechnology, Acharya Nagarjuna University, Guntur City, Andhra Pradesh, India; 4 Department of Genetics and Molecular Biology, University of Panama, Panama City, Panama; 5 INDICASAT-AIP, City of Knowledge, Clayton, Panama City, Panama; University of Georgia, United States of America

## Abstract

The Human immunodeficiency virus type-1 (HIV-1) epidemic in the Caribbean region is mostly driven by subtype B; but information about the pattern of viral spread in this geographic region is scarce and different studies point to quite divergent models of viral dissemination. In this study, we reconstructed the spatiotemporal and population dynamics of the HIV-1 subtype B epidemic in the Caribbean. A total of 1,806 HIV-1 subtype B *pol* sequences collected from 17 different Caribbean islands between 1996 and 2011 were analyzed together with sequences from the United States (*n* = 525) and France (*n* = 340) included as control. Maximum Likelihood phylogenetic analyses revealed that HIV-1 subtype B infections in the Caribbean are driven by dissemination of the pandemic clade (B_PANDEMIC_) responsible for most subtype B infections across the world, and older non-pandemic lineages (B_CAR_) characteristics of the Caribbean region. The non-pandemic B_CAR_ strains account for >40% of HIV-1 infections in most Caribbean islands; with exception of Cuba and Puerto Rico. Bayesian phylogeographic analyses indicate that B_CAR_ strains probably arose in the island of Hispaniola (Haiti/Dominican Republic) around the middle 1960s and were later disseminated to Trinidad and Tobago and to Jamaica between the late 1960s and the early 1970s. In the following years, the B_CAR_ strains were also disseminated from Hispaniola and Trinidad and Tobago to other Lesser Antilles islands at multiple times. The B_CAR_ clades circulating in Hispaniola, Jamaica and Trinidad and Tobago appear to have experienced an initial phase of exponential growth, with mean estimated growth rates of 0.35–0.45 year^−1^, followed by a more recent stabilization since the middle 1990s. These results demonstrate that non-pandemic subtype B lineages have been widely disseminated through the Caribbean since the late 1960s and account for an important fraction of current HIV-1 infections in the region.

## Introduction

Globally, an estimated 34 million people were infected with the human immunodeficiency virus-type 1 (HIV-1), the aetiologic agent of acquired immunodeficiency syndrome (AIDS), at the end of 2012 [1]. The Caribbean is one of the most severely affected regions in the world after Sub-Saharan Africa. About 250,000 people (1.0% of the adult population) were living with HIV-1 in the Caribbean in 2012, 78% of whom were reported in Hispaniola, the island shared by the Dominican Republic and Haiti [1]. HIV prevalence greatly varies among countries ranging from <0.1% in Cuba to over 2% in the Bahamas and Haiti [1]. The first AIDS cases were recognized in Haiti in 1978–1979 [2] and in other Caribbean countries in 1982–1984 [3], [4], [5]. The main mode of HIV transmission in the region is heterosexual sex [6].

The subtype B dominates the HIV-1 epidemic in most Caribbean islands [7], [8], [9], [10], [11], [12], [13], [14], with exception of Cuba where several non-B genetic forms are collectively more prevalent [15], [16], [17], [18], [19], [20]. Genetic evidence suggests that the HIV-1 subtype B was introduced from Central Africa into America through Haiti around the middle 1960s, coinciding with the return of many Haitian professionals who worked in the Democratic Republic of Congo [21]. According to that study, one subtype B strain was disseminated from Haiti to the United States (US) around 1969 and from the US to the rest of the world, establishing a “B_PANDEMIC_” clade. Other subtype B lineages, here called “B_CAR_” clades, remain mostly restricted to Haiti and neighboring Caribbean islands. The study of Gilbert *et al* (2007), however, analyzed a very low number of HIV-1 subtype B Caribbean sequences (Haiti = 11 and Trinidad and Tobago = 11) and the relative prevalence of the B_PANDEMIC_ and B_CAR_ clades across different Caribbean islands remains largely unknown.

A more recent study that analyzed 836 HIV-1 subtype B *pol* gene sequences from 13 different Caribbean countries, suggests a very different model of viral dispersion [22]. The study indicates that most of the current subtype B variability in the Caribbean islands was generated since the early 1980s and onwards and that the virus was mainly disseminated from Antigua and Puerto Rico following two distinguishable routes. During the 1980s the virus would have jumped from Antigua to other Lesser Antilles (Barbados, Dominica, Grenada, Trinidad and Tobago, St. Lucia and St. Vincent), Bahamas, Haiti and Jamaica. In the same period, the virus would have spread from Puerto Rico to Cuba, Jamaica, Haiti and Dominican Republic. The authors proposed that B_CAR_ clades early disseminated from Haiti resulted in dead-end infections and that a second introduction of the B_PANDEMIC_ clade from the US through Puerto Rico during the 1980s generated the actual epidemic in the Caribbean region. Other phylogenetic studies, however, reveal that some subtype B *pol* Caribbean sequences recently isolated branched within the B_PANDEMIC_ clade while other branched at more basal positions within the subtype B phylogeny [23], [24], [25], thus suggesting a continuous circulation of non-pandemic B_CAR_ clades in the Caribbean region.

The objective of this study was to estimate the current prevalence of the B_PANDEMIC_ and B_CAR_ clades in the Caribbean islands and to reconstruct the spatiotemporal dynamics of dissemination of the HIV-1 subtype B in the region. For this, we used a comprehensive dataset of HIV-1 subtype B *pol* sequences (*n* = 1,806) isolated from 17 different Caribbean islands between 1996 and 2011. These Caribbean sequences were combined with subtype B sequences from the US (*n* = 525) and France (*n* = 340) and subjected to Maximum Likelihood and Bayesian phylogeographic analyses.

## Materials and Methods

### HIV-1 subtype B *pol* sequence dataset

We retrieved all HIV-1 subtype B *pol* sequences with known sampling date from the Caribbean, US and France that covered the entire protease and partial reverse transcriptase (PR/RT) regions (nt 2253–3260 relative to the HXB2 clone) that were available at the Los Alamos HIV Database (http://www.hiv.lanl.gov) by June 2013. Additional HIV-1 subtype B *pol* sequences available in the same database, but only covering part of the RT (nt 2673–3203 relative to the HXB2 clone) were also downloaded from Barbados, Guadeloupe, Haiti, Martinique, Puerto Rico, and US Virgin Islands. Only one sequence per subject was selected and those sequences containing frameshift mutations were removed from the alignment. For the analyses to run in a reasonable time, some sequences from the US, the most overrepresented country in our dataset with about 10,000 sequences, were removed. In order to generate a “non-redundant” subset representative of the HIV-1 subtype B diversity in the US, highly similar (identity ≥95%) sequences from this country were clustered with the CD-HIT program [26] using an online web server [27] and only one sequence per cluster was selected. This resulted in a final data set of 2,671 subtype B *pol* sequences isolated from the Caribbean (*n* = 1,806), US (*n* = 525), and France (*n* = 340) between 1982 and 2011 (Table 1). The number and geographic representation of subtype B Caribbean sequences available from other genomic regions was very limited, thus we decided to focus on the *pol* gene only. The subtype assignment of all sequences included was confirmed using the REGA HIV subtyping tool v.2 [28] and by performing Maximum Likelihood (ML) phylogenetic analyses (see below) with HIV-1 group M subtype reference sequences. Sequences were aligned and all sites associated with major antiretroviral drug resistance in PR (30, 32, 46, 47, 48, 50, 54, 76, 82, 84, 88 and 90) and RT (41, 65, 67, 69, 70, 74, 100, 101, 103, 106, 115, 138, 151, 181, 184, 188, 190, 210, 215, 219 and 230) detected in at least two sequences were excluded. All alignments are available from the authors upon request.

**Table 1:** HIV-1 subtype *pol* sequences.

Region	Country	*N* (PR/RT)	*N* (RT)	Sampling time
Caribbean Greater Antilles	Cuba	319	-	1999–2011
	Puerto Rico	6	285	2005–2007
	Dominican Republic	168	-	2002–2011
	Jamaica	146	-	2001–2010
	Haiti	16	15	2004–2005
Caribbean Lesser Antilles	Martinique	-	452	1997–2006
	Guadeloupe	-	243	1999–2004
	US/Virgin Islands	-	54	2003–2004
	Trinidad and Tobago	52	-	2000–2003
	Others*	25	14	1996–2000
Caribbean Bahamas	Bahamas	11	-	2004
North America	US	525	-	1982–2010
Europe	France	340	-	1983–2008

*Antigua and Barbuda (*n* = 7), Barbados (*n* = 14), Dominica (*n* = 3), Grenada (*n* = 4), Montserrat (*n* = 1), Saint Lucia (*n* = 4) and Saint Vincent and the Grenadines (*n* = 6).

### Phylogenetic analysis

Maximum Likelihood (ML) phylogenetic trees were inferred under the GTR+I+Г_4_ nucleotide substitution model selected using the jModeltest program [29]. The ML trees were reconstructed with the PhyML program [30] using an online web server [31]. Heuristic tree search was performed using the SPR branch-swapping algorithm and the reliability of the obtained topology was estimated with the approximate likelihood-ratio test (*aLRT*) [32] based on the Shimodaira-Hasegawa-like procedure. The ML trees were visualized using the FigTree v1.4.0 program [33].

### Analysis of the spatiotemporal dispersion pattern

The evolutionary rate, the age of the most recent common ancestor (*T*_MRCA_) and the spatial diffusion pattern of HIV-1 B_CAR_ clades were jointly estimated using a Bayesian Markov Chain Monte Carlo (MCMC) approach implemented in BEAST v1.8 [34], [35] with BEAGLE to improve run-time [36]. Analyses were performed using the GTR+I+Г_4_ nucleotide substitution model and a relaxed uncorrelated lognormal molecular clock model [37]. The mean evolutionary rates previously estimated for the subtype B *pol* gene (1.7–3.0×10^−3^ subst./site/year) [24], [38], [39], [40] were incorporated as an informative prior interval. Migration events were reconstructed using a reversible discrete phylogeography model [41] with a CTMC rate reference prior [42]. The most relevant migration pathways and the number of viral migrations between locations were estimated by using the Bayesian stochastic search variable selection (BSSVS) [39] and the ‘Markov jump’ counts [43], [44], [45] approaches, respectively. Three MCMC chains were run for 4×10^8^ generations and then combined. Effective Sample Size (ESS) and 95% Highest Probability Density (HPD) values were inspected using Tracer v1.6 [46] to asses convergence and uncertainty of parameter estimates. The maximum clade credibility (MCC) tree was summarized with TreeAnnotator v1.8 and visualized with FigTree v1.4.0. Migratory events were summarized using the SPREAD application [47].

### Reconstruction of demographic history

The mode and rate of population growth of some country-specific HIV-1 B_CAR_ clades was also estimated using the BEAST v1.8 software. Changes in effective population size through time were initially estimated using a Bayesian Skyline coalescent tree prior [48] and estimates of the population growth rate were subsequently obtained using the parametric model (logistic, exponential or expansion) that provided the best fit to the demographic signal contained in datasets. Comparison between demographic models was performed using the log marginal likelihood (ML) estimation based on path sampling (PS) and stepping-stone sampling (SS) methods [49]. The MCMC analyses were run for 100–200 million generations. Convergence of parameters and uncertainty in parameter estimates were assessed as described in the previous paragraph.

## Results

### Evidence of co-circulation of B_PANDEMIC_ and B_CAR_ clades in the Caribbean

**Figure 1:**
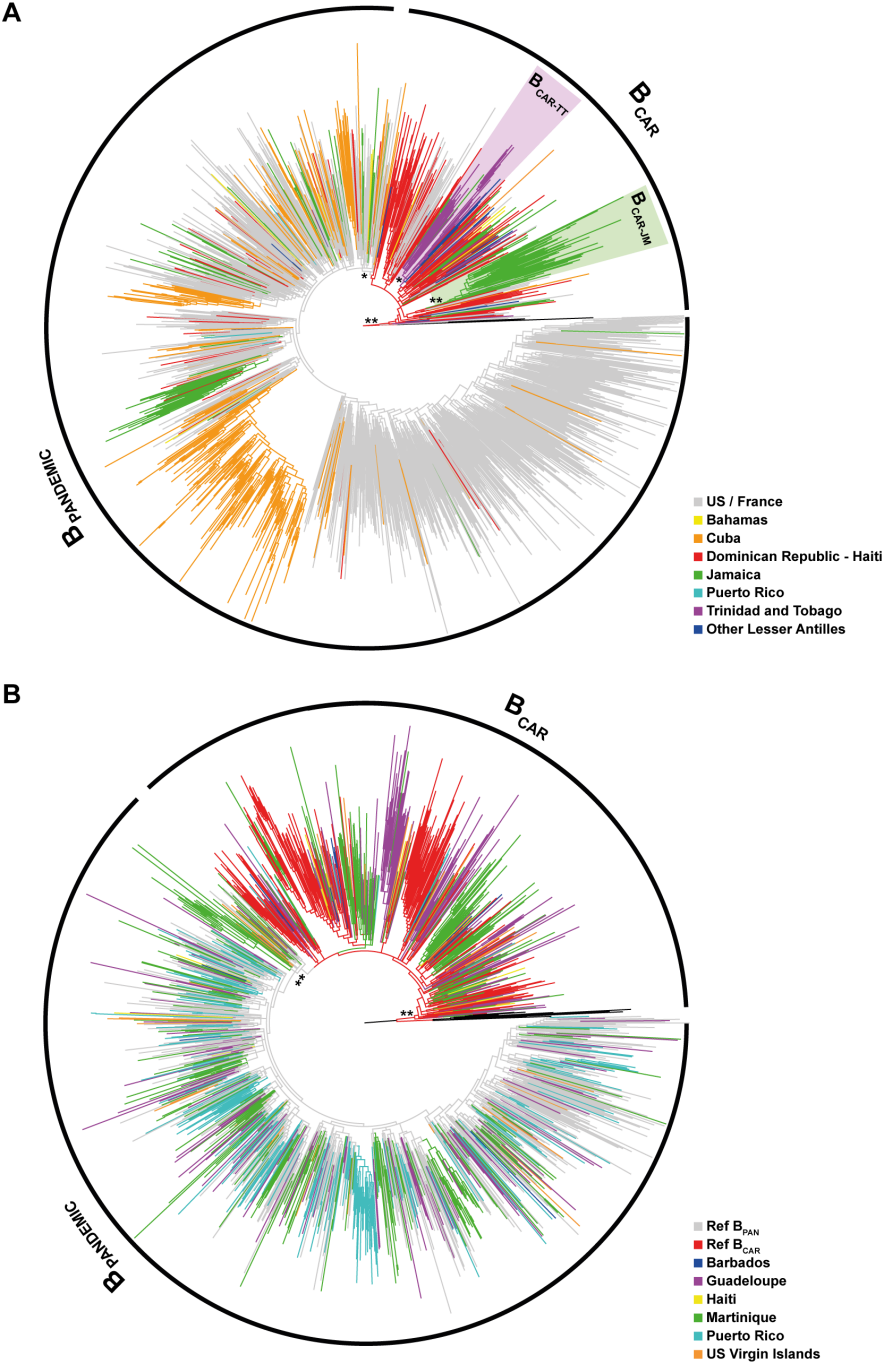
ML phylogenetic tree. A) HIV-1 subtype B *pol* PR/RT sequences (∼1,000 nt) circulating in the Caribbean (*n* = 743), US (*n* = 525), and France (*n* = 340); B) HIV-1 subtype B *pol* RT (∼600 nt) sequences from Barbados (*n* = 14), Guadeloupe (*n* = 243), Haiti (*n* = 15), Martinique (*n* = 452), Puerto Rico (*n* = 285), US Virgin Islands (*n* = 54) and representative sequences of the B_PANDEMIC_ (US = 165, France = 135) and the B_CAR_ (Caribbean = 200) clades. Branches are colored according to the geographic origin of each sequence as indicated at the legend (bottom right). Arcs indicate the B_PANDEMIC_ and B_CAR_ clades. Purple and green shaded boxes highlight the position of the Caribbean clades B_CAR-TT_ and B_CAR-JM_. The *a*LRT support values are indicated at key nodes: *(0.81–0.90), **(0.91–1). Trees were rooted using HIV-1 subtype D reference sequences. The branch lengths are drawn to scale with the bar at the bottom indicating nucleotide substitutions per site.

In order to estimate the relative prevalence of pandemic (B_PANDEMIC_) and non-pandemic (B_CAR_) subtype lineages in the Caribbean, subtype B *pol* (PR/RT) sequences from different Caribbean islands (*n* = 743) were combined with subtype B sequences from the US (*n* = 525) and France (*n* = 340), two countries with epidemics dominated by the B_PANDEMIC_ clade. The ML analysis revealed that, as expected, most HIV-1 subtype B sequences from the US (89.7%) and France (99.7%) branched in a well supported (a*LRT* = 0.85) monophyletic subgroup (the B_PANDEMIC_ clade) nested within basal non-pandemic lineages (the B_CAR_ clades) that branched closer to the subtype B root (Fig. 1A). Most of the deepest B_CAR_ lineages were traced to the Caribbean region (83.9%), although their prevalence greatly varies across islands ranging from 96.1% in Trinidad and Tobago to 0.9% in Cuba (Table S1). This analysis also revealed that B_CAR_ sequences from the Dominican Republic and Haiti, two nations located in the island of Hispaniola, were highly intermixed with each other and occupied the deepest branches in the subtype B phylogeny; whereas most B_CAR_ sequences from Jamaica and Trinidad and Tobago branched in two country-specific subclades, B_CAR-JM_ (a*LRT* = 0.92) and B_CAR-TT_ (a*LRT* = 0.84), that were nested among basal sequences from Hispaniola (Fig. 1A). The number of B_CAR_ sequences from other Caribbean islands was too small to evaluate their clustering pattern.

**Figure 2:**
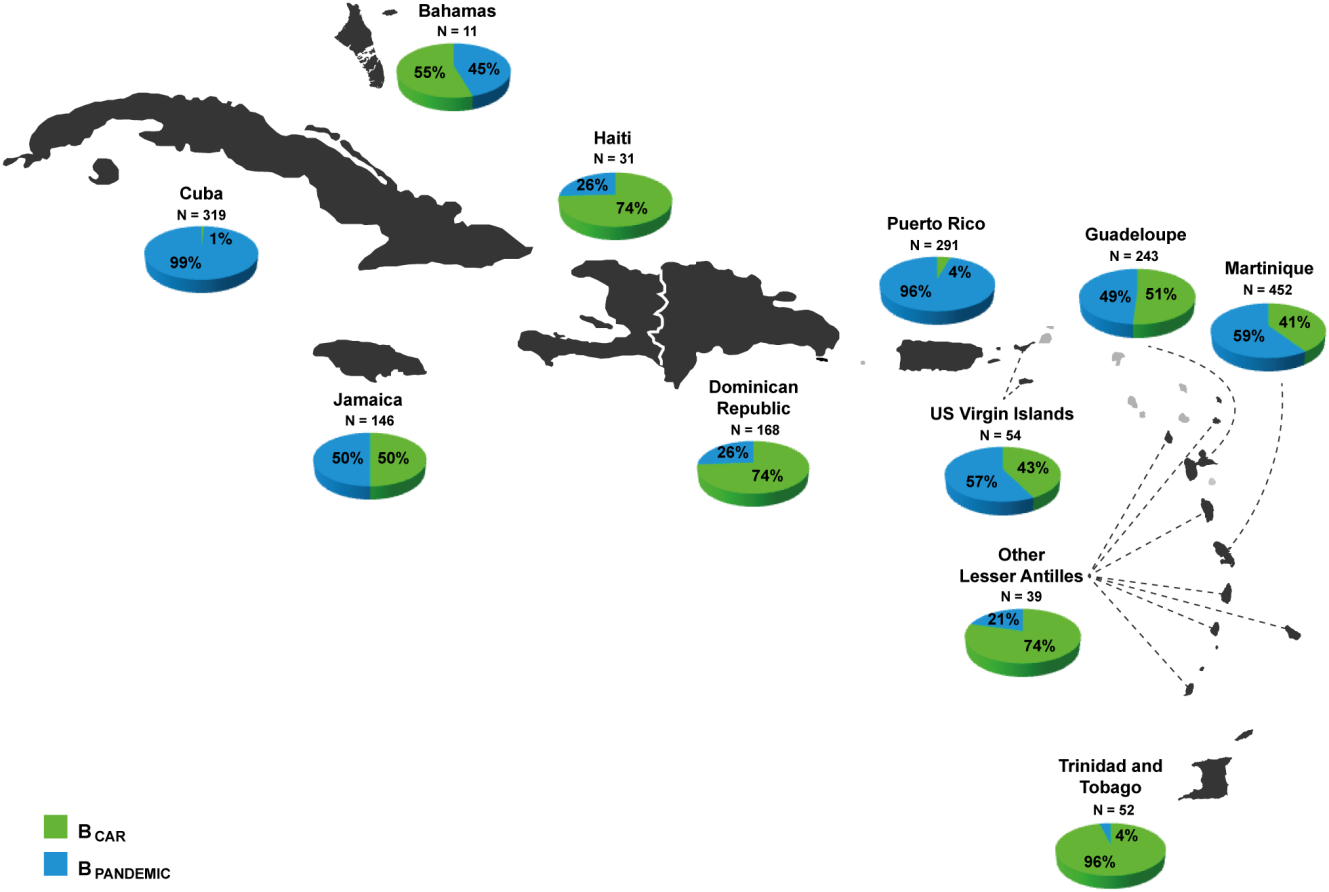
Estimated proportion of B_CAR_ and B_PANDEMIC_ clades among HIV-1 subtype B infected individuals from different Caribbean countries according to the ML analyses. The total number of sequences analyzed in each locality is indicated.

In order to obtain more accurate estimates of the prevalence of B_PANDEMIC_ and B_CAR_ clades in the Caribbean region, we performed a second ML analysis that combined shorter subtype B *pol* (RT) Caribbean sequences (*n* = 1,063) from countries poorly represented in the PR/RT dataset (Barbados, Guadeloupe, Haiti, Martinique, Puerto Rico and US Virgin Islands) with reference sequences representative of the B_PANDEMIC_ (US/France = 300) and B_CAR_ (Caribbean = 200) clades selected from the previous analysis. The new ML analysis confirmed the complete segregation of the B_PANDEMIC_ and B_CAR_ reference sequences and the co-circulation of both pandemic and non-pandemic lineages in all Caribbean islands (Fig. 1B); although their relative prevalence greatly vary across countries (Table S1). These results support the existence of three major HIV-1 molecular epidemiologic scenarios in the Caribbean region (Fig. 2). The first one, represented by Haiti, Dominican Republic, Trinidad and Tobago and some other Lesser Antilles, is characterized by the predominance (>70%) of non-pandemic B_CAR_ clades. The second one, represented by Jamaica, Guadeloupe, Martinique, US Virgin Islands and probably the Bahamas, is characterized by roughly similar frequency of both B_PANDEMIC_ and B_CAR_ clades. The third one is represented by Cuba and Puerto Rico where the vast majority (>97%) of subtype B sequences belong to the B_PANDEMIC_ clade.

### Spatiotemporal dispersal pattern of the HIV-1 B_CAR_ clades in the Caribbean

The origin and spatiotemporal dynamics of non-pandemic subtype B Caribbean lineages was then reconstructed using a Bayesian phylogeographic analysis. The B_CAR_ *pol* (PR/RT) sequences from the Dominican Republic, Haiti, Jamaica, and the Lesser Antilles were classified into nine discrete locations: Hispaniola (*n* = 136), Jamaica (*n* = 73), Trinidad and Tobago (*n* = 52), Antigua and Barbuda (*n* = 4), Dominica (*n* = 2), Grenada (*n* = 3), Montserrat (*n* = 1), Saint Lucia (*n* = 4) and Saint Vincent and the Grenadines (*n* = 6), and combined with subtype D sequences from the Democratic Republic of Congo (DRC). The estimated evolutionary rate of the HIV-1 B/D *pol* dataset was 1.74×10^−3^ (95% HPD: 1.70×10^−3^–1.80×10^−3^) substitutions/site per year and the corresponding coefficient of rate variation was 0.27 (95% HPD: 0.22–0.32), thus supporting the selection of a relaxed molecular clock model.

**Figure 3:**
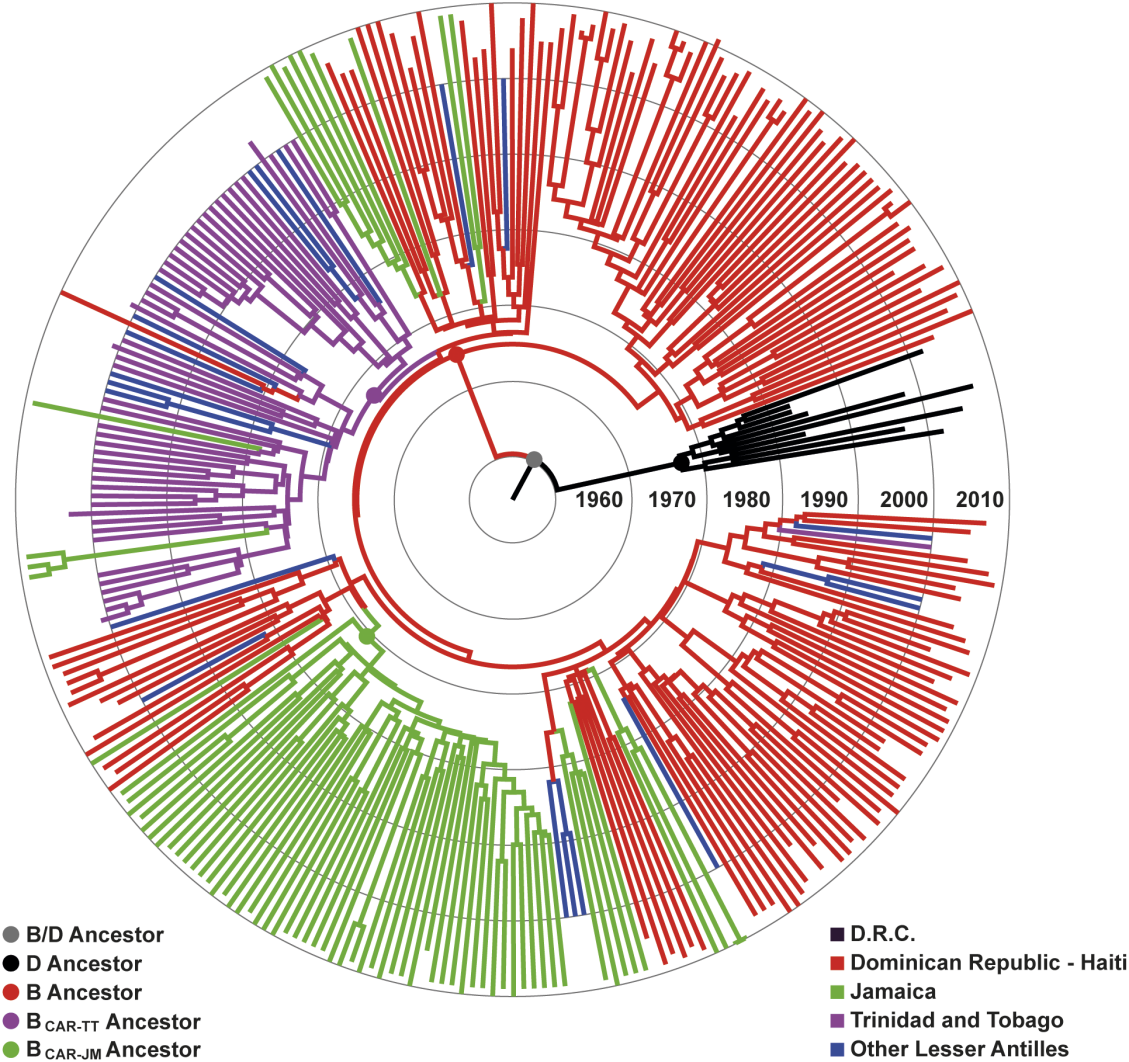
Time-scaled Bayesian MCMC tree of the HIV-1 B_CAR_ lineages from the Caribbean and subtype D reference sequences from the Democratic Republic of Congo (DRC). Branches are colored according to the most probable location state of their descendent nodes. Color code is indicated at the legend at bottom right. Colors circles indicate the positions of nodes corresponding to the most recent common ancestors of major clades, as indicated at the legend at bottom left. Branch lengths are depicted in units of time (years). The tree was automatically rooted under the assumption of a relaxed molecular clock.

**Figure 4:**
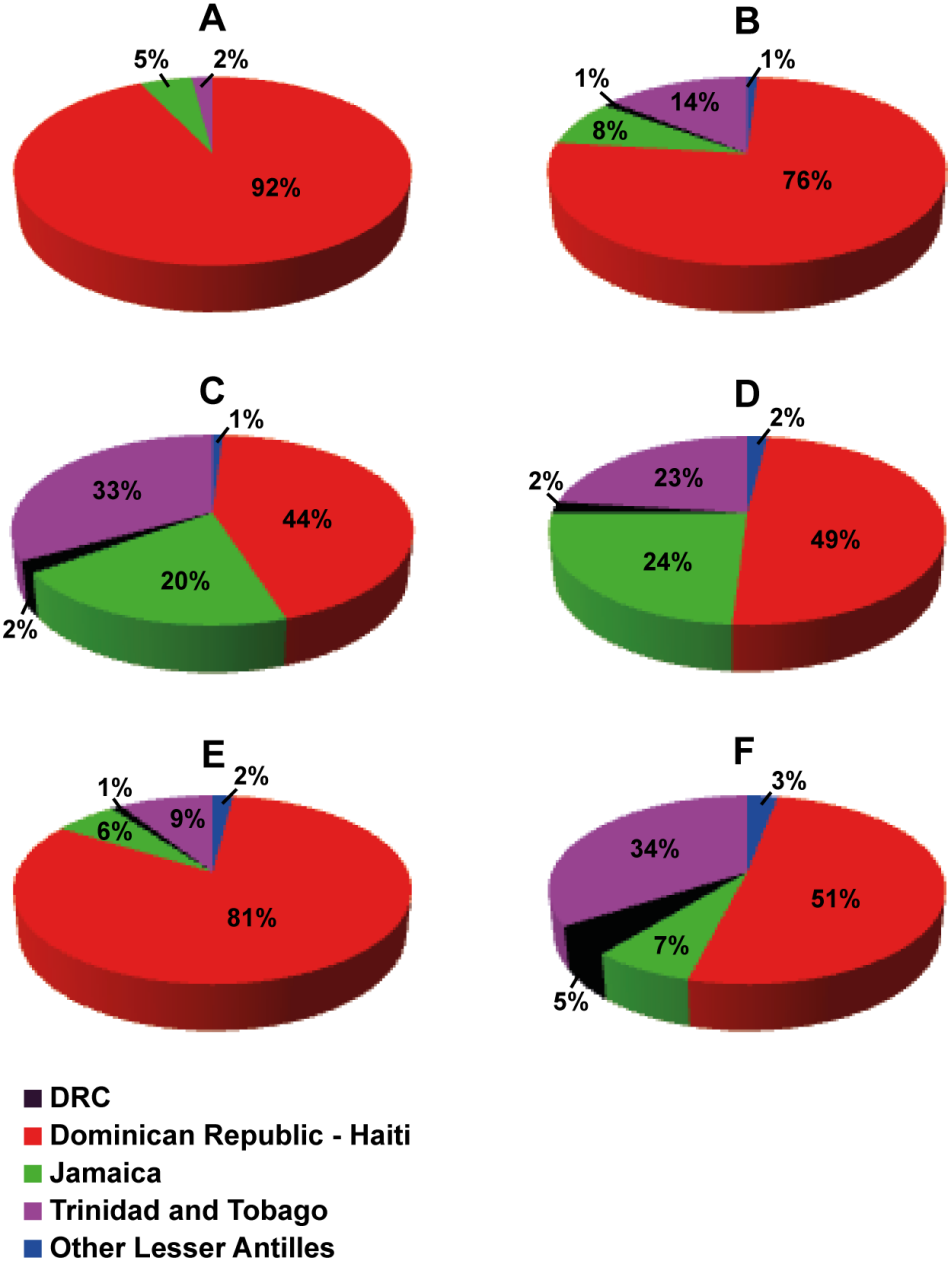
Root location of the HIV-1 subtype B. Graphics depict the location state posterior probability distributions at the root of the subtype B_CAR_ clades at the Bayesian MCC trees obtained from complete dataset (A) and country “balanced” sub-sets (B to G) from the Caribbean region. Color code is indicated at the legend at bottom left.

Consistent with the previous ML analysis, the overall topology of the Bayesian MCC tree showed that most sequences from Jamaica and Trinidad and Tobago branched in two country-specific subclades that were nested within the basal sequences from Hispaniola (Fig. 3). The mean estimated T_MRCA_ for the major HIV-1 lineages were as follows: subtypes B/D = 1952, subtype D = 1965, subtype B = 1964, B_CAR-TT_ = 1969, and B_CAR-JM_ = 1971; very similar to that previously described by Gilbert *et al* (2007) (Table 2). The most probable root location of the HIV-1 subtype B ancestor was placed in Hispaniola (posterior state probability [*PSP*] = 0.92) (Figs. 3 and 4A). Because most of the HIV-1 B_CAR_ sequences included in our dataset were from Hispaniola (48.7%), we generated five “balanced” subsets containing up to 25 sequences from each location (Table S2). Hispaniola was pointed out as the most probable root location of the B clade in all “balanced” subsets, although the support was lower than that obtained for the complete dataset (*PSP* = 0.44–0.81) (Figs. 4B to 4F).

**Table 2:** Bayesian time-scale estimates for the origin of HIV-1 subtypes B and D.

Clade	Current T_MRCA_ estimates	Previous T_MRCA_ estimates*
Subtypes B/D	1952 (1943–1960)	1954 (1946–1961)
Subtype D	1965 (1958–1971)	1966 (1961–1971)
Subtype B	1964 (1959–1969)	1966 (1962–1970)
B_CAR-TT_	1969 (1966–1973)	1973 (1970–1976)
B_CAR-M_	1971 (1967–1975)	-

*Estimated by Gilbert *et al* (2007).

**Figure 5:**
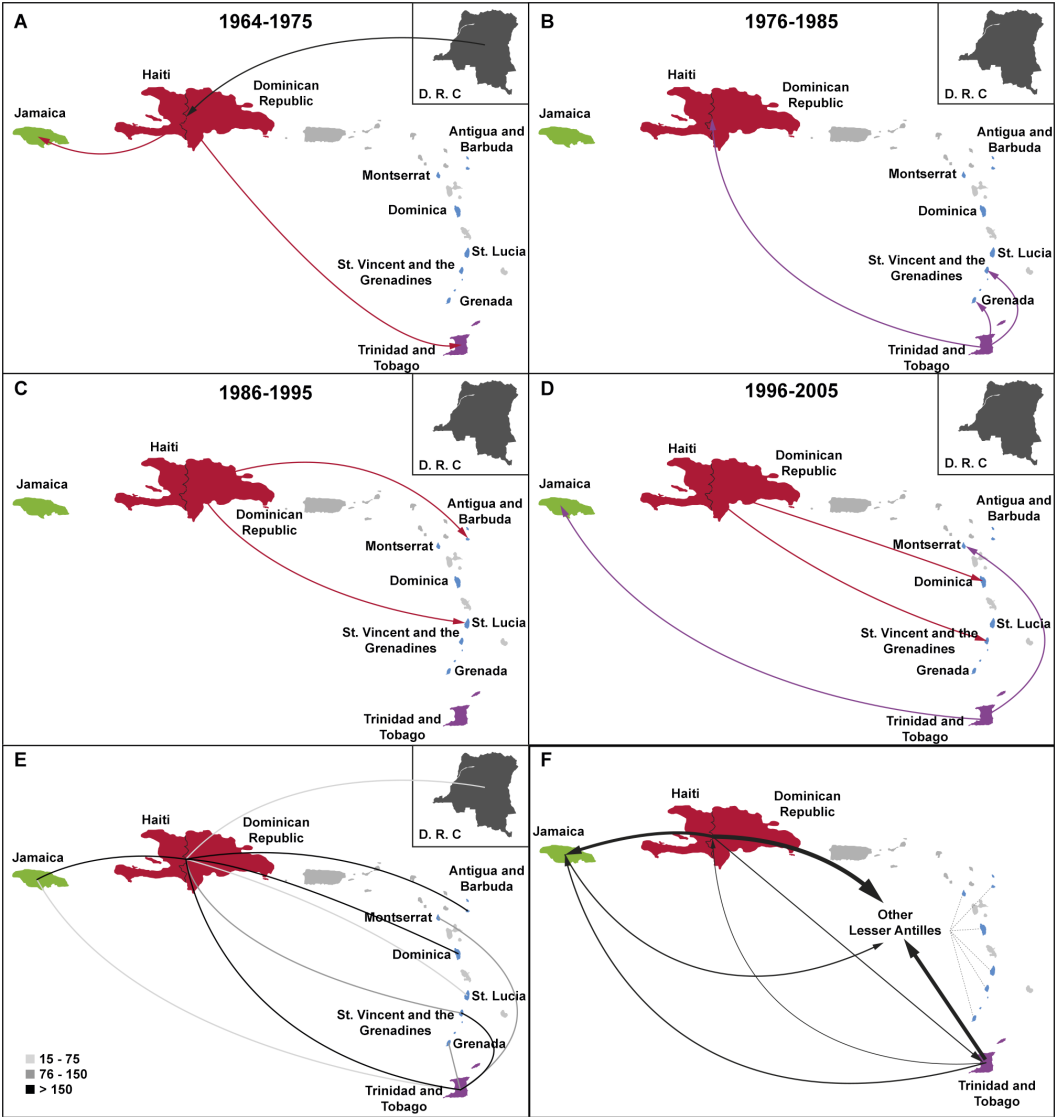
Spatiotemporal dynamics of dissemination of non-pandemic HIV-1 B_CAR_ clades in the Caribbean region. A–D) Viral migration events occurred between 1964 and 2005 are indicated. Lines between locations represent branches in the Bayesian MCC tree along which location transitions occurred. The line’s color informs the source location and only the earliest transitions between each location pair were represented. E) Most significant epidemiological links of the dissemination process of B_CAR_ clades. Only epidemiological links supported by Bayes factor rates >3 are displayed. The values of Bayes factor rates are indicated by a color gradient that ranges from light to dark grey as indicated at the legend (up left). F) Viral migrations among locations as measured using ‘Markov jump’ counts. The width of the arrows is proportional to the corresponding mean estimated number of viral transitions between locations. No arrows were displayed when the mean estimated number of transitions was below one.

Reconstruction of viral migrations across time from the complete dataset revealed a rapid dissemination of B_CAR_ clades across the Caribbean region (Figs. 5A–5D). After the introduction of HIV-1 subtype B into Hispaniola around the middle 1960s, non-pandemic B_CAR_ lineages were independently disseminated to Trinidad and Tobago and Jamaica around the late 1960s and the early 1970s, respectively. Those early introductions seeded secondary outbreaks in Trinidad and Tobago and Jamaica that resulted in the origin of the B_CAR-TT_ and B_CAR-JM_ clades. Several independent transmissions of non-pandemic B_CAR_ clades from Hispaniola to Jamaica (*n* = 7), Trinidad and Tobago (*n* = 1) and the other Lesser Antilles (*n* = 8) were detected from the late 1970s onwards. In the same time period, our data indicates that the B_CAR-TT_ clade was also independently disseminated from Trinidad and Tobago to other Lesser Antilles (*n* = 6), Jamaica (*n* = 2) and Hispaniola (*n* = 1). In contrast, we found no evidence of dissemination of the B_CAR-JM_ clade out of Jamaica.

The Bayes factor tests for significant nonzero rates, supports epidemiological linkage between DRC and Hispaniola; between Hispaniola and Jamaica/Trinidad and Tobago/Antigua and Barbuda/Dominica/St. Lucia/St. Vincent and the Grenadines; and between Trinidad and Tobago and Jamaica/Grenada/Montserrat/St. Vincent and the Grenadines (Fig. 5E and Table S3). The Markov jump counts analysis indicates that the most viral transitions between epidemiologically linked locations were from Hispaniola to Jamaica and the Lesser Antilles (except Trinidad and Tobago), and from Trinidad and Tobago to the other Lesser Antilles (Fig. 5F and Table S4). Lower numbers of viral migrations were detected between Jamaica/Trinidad and Tobago and between Hispaniola/Trinidad and Tobago. The highest net viral migration flux (efflux minus influx) was for Hispaniola (11.45), followed by Trinidad and Tobago (7.30), Jamaica (−4.05), and other Lesser Antilles (−14.70).

### Demographic history of the HIV-1 B_CAR_ clades in the Caribbean

**Figure 6:**
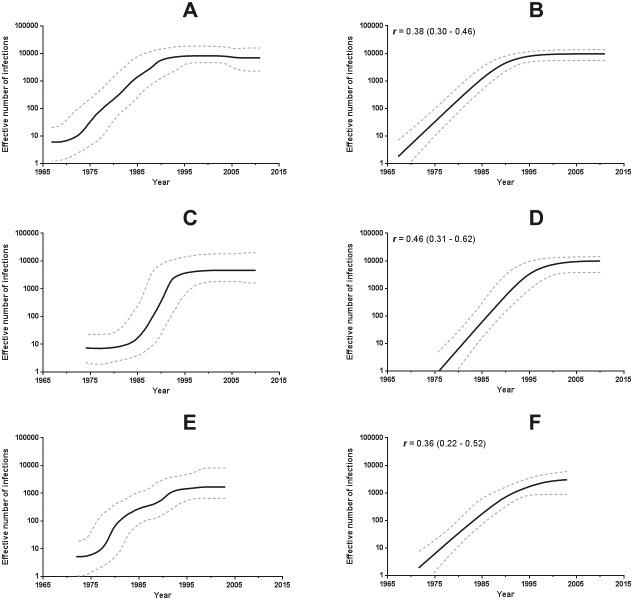
Demographic history of the HIV-1 B_CAR_ (A, B), B_CAR-JM_ (C, D), and B_CAR-TT_ (E, F) clades circulating in Hispaniola, Jamaica and Trinidad and Tobago, respectively. Effective number of infections (y-axis; log10 scale) through time (x-axis; calendar years) estimated using Bayesian skyline (A, C, E) and logistic (B, D, F) growth coalescent model. Median estimates of the effective number of infections (solid line) and 95% HPD intervals of the estimates (dashed lines) are shown in each graphic. The median growth rate (with the corresponding 95% credibility interval in parenthesis) of each clade estimated under logistic growth model is indicated in the upper left corner.

We reconstructed the population dynamic pattern of the HIV-1 B_CAR_ clades from Hispaniola (*n* = 136), the B_CAR-TT_ clade from Trinidad and Tobago (*n* = 49) and the B_CAR-JM_ clade from Jamaica (*n* = 50). Substitution rate and T_MRCA_ estimates obtained in the previous Bayesian analysis were used as prior intervals for demographic reconstructions. The Bayesian skyline plot (BSP) coalescent analysis suggests that all Caribbean clades experienced an initial phase of fast exponential growth followed by a more recent decline in growth rate since the middle 1990s, consistent with a model of logistic growth (Fig. 6). The log ML for the logistic, exponential, and expansion growth models were then calculated using both PS and SS methods. The model of logistic population growth was strongly supported over the other for all HIV-1 Caribbean clades (log BF >3) (Table 3). According to the logistic growth coalescent model, the Caribbean clades exhibited mean initial growth rates that range from 0.36 year^−1^ to 0.46 year^−1^, with great overlap of the 95% HPD intervals (Fig. 6).

**Table 3:** Best fit demographic model for different HIV-1 subtype B_CAR_ clades.

B_CAR_ Clade	Model	PS(Log ML)	Modelscompared	Log BF	SS(Log ML)	Modelscompared	Log BF
B_DO-HT_	Log	−15090.0	-	-	−15092.0	-	-
	Expo	−15142.1	Log/Expo	52.1	−15143.8	Log/Expo	51.8
	Expa	−15144.3	Log/Expa	54.3	−15145.7	Log/Expa	53.7
B_JA_	Log	−6873.6	-	-	−6874.0	-	-
	Expo	−6886.4	Log/Expo	12.8	−6886.6	Log/Expo	12.6
	Expa	−6893.9	Log/Expa	20.3	−6894.1	Log/Expa	20.1
B_TT_	Log	−6034.8	-	-	−6034.9	-	-
	Expo	−6037.7	Log/Expo	2.9	−6038.0	Log/Expo	3.1
	Expa	−6044.0	Log/Expa	9.2	−6044.1	Log/Expa	9.2

Log marginal likelihood (ML) estimates for the logistic (Log), exponential (Expo) and expansion (Expa) growth demographic models obtained using the path sampling (PS) and stepping-stone sampling (SS) methods. The Log Bayes factor (BF) is the difference of the Log ML between of alternative (H1) and null (H0) models (H1/H0). Log BF >3 indicate that model H1 is more strongly supported by the data than model H0.

## Discussion

This study demonstrates that the HIV-1 subtype B epidemic in the Caribbean is driven by dissemination of the pandemic clade (B_PANDEMIC_), responsible for most subtype B infections across the world, as well as non-pandemic lineages (B_CAR_). The relative prevalence of the different subtype B clades greatly varies among countries, giving rise to three major epidemiologic scenarios: 1) islands where B_CAR_ lineages are predominant (Haiti, Dominican Republic and some Lesser Antilles); 2) islands where epidemic is mainly driven by the B_PANDEMIC_ clade (Cuba and Puerto Rico); and 3) islands where both B_PANDEMIC_ and B_CAR_ clades circulates at roughly similar proportions (Jamaica, Guadeloupe, Martinique, US Virgin Islands and the Bahamas).

The differential spreading of B_PANDEMIC_ and B_CAR_ clades across Caribbean islands probably resulted from the combination of chance effects and socio-ecological factors. The HIV-1 epidemic in most Caribbean countries is mainly driven by heterosexual sex [6], with exception of Cuba and Puerto Rico where the epidemic is driven primarily by populations of men having sex with men (MSM) [20], [50] and injection drug users (IDUs) [6], [51], respectively. As a commonwealth of the United States, an extensive migration/travel of Puerto Rican IDUs between Puerto Rico and New York has been reported [52], [53]. It is also interesting to note that the origin of the major HIV-1 subtype B Cuban clades, estimated around the early 1990s [24], coincides with an abrupt increase in the number of tourists from North America and Europe that visited Cuba [54]. These singular socio-ecological factors may have fueled the chance introduction of the B_PANDEMIC_ clade in the Cuban MSM and Puerto Rican IDUs, which explains the preferential dissemination of this subtype B variant in those islands.

Our study indicates that HIV-1 subtype B likely entered in the Americas through the island of Hispaniola around the middle 1960s, in agreement with the model proposed by Gilbert *et al* (2007). HIV sequences from Haiti and the Dominican Republic were phylogenetically intermixed among each other, consistent with the geographic proximity and intense human mobility between those countries [55], making it difficult to determine which country of Hispaniola was the entrance point for this subtype. Notably, the HIV prevalence in the Dominican Republic is particularly high (5%) among the Haitian-origin communities of the *bateyes* [56]; suggesting that the Dominican Republic epidemic has been partially driven by links to Haiti. This epidemiological context combined with historical data that also links Haiti to the DRC in the 1960s [21], supports the notion that subtype B was most likely first introduced into Haiti and later disseminated to the Dominican Republic at multiple times.

Non-pandemic B_CAR_ strains probably started to spread from Haiti and/or Dominican Republic to Trinidad and Tobago and Jamaica between the late 1960s and the early 1970s, seeding secondary outbreaks that gave rise to local non-pandemic Caribbean clades here called B_CAR-TT_ and B_CAR-JM_. The B_CAR-TT_ clade was previously described by Gilbert *et al* (2007) and comprises 94% of subtype B sequences from Trinidad and Tobago here included, supporting that this epidemic mainly resulted from dissemination of a single founder non-pandemic strain [7], [21], [57]. The B_CAR-JM_ clade comprises 34% of subtype B sequences from Jamaica, indicating a polyphyletic origin of the Jamaican epidemic. Indeed, the second largest Jamaican-specific clade that comprises 19% of subtype B sequences from this country was nested among B_PANDEMIC_ strains. The B_CAR_ strains may have also seeded secondary HIV epidemics in other Lesser Antilles islands, although more sequences should be analyzed to confirm this observation.

While non-pandemic B_CAR_ clades started to spread some years earlier or around the same time as the B_PANDEMIC_ clade, the final outcome of each subtype B lineage was very different. Whereas the B_PANDEMIC_ lineage was disseminated across the world, the B_CAR_ clades seems mainly confined to the Caribbean region. HIV-1 B_CAR_ clades circulate in some Caribbean countries (Haiti, Bahamas, Barbados, Trinidad and Tobago and Jamaica) with high HIV prevalence rates (>1%), thus arguing against the hypothesis of a low epidemic potential of non-pandemic B clades. Our results also revealed that B_CAR_ clades moved from the Caribbean into the US at multiple occasions (Fig. 1A); but most introductions failed to ignite significant outbreaks. This suggests that socio-ecological factors have probably played a key role in the final outcome of the different subtype B lineages. The chance introduction of the B_PANDEMIC_ ancestor into a group of individuals with high rates of partner exchanges and living in a globally interconnected country like the US may explain the successful worldwide dissemination of that viral clade.

The first AIDS cases reported in Jamaica and Trinidad and Tobago in the early 1980s were among MSM who engaged in sex with North American MSM [58], [59]. This observation reinforced the hypothesis that HIV-1 was first introduced into the Caribbean islands via homosexual contact with North American foreigners in the late 1970s or early 1980s [7], [10], [58], [59]. Our results confirm that the B_PANDEMIC_ clade has been introduced and disseminated in most Caribbean islands. The firsts AIDS cases described in Jamaica and Trinidad and Tobago; however, were most probably linked to the transmission of B_CAR_ viruses disseminated out from Hispaniola since the late 1960s. The mean estimated T_MRCA_ of the B_CAR-TT_ (1969) and B_CAR-JM_ (1973) clades coincide with that estimated for the B_PANDEMIC_ clade (1969) [21]; suggesting that HIV epidemics in Jamaica, Trinidad and Tobago and the US started around the same time which is fully consistent with the nearly simultaneous description of the firsts AIDS cases in the those countries at the early 1980s.

The results presented here also clearly contrast with the hypothesis that B_CAR_ clades early disseminated from Haiti resulted in dead-end infections in the Caribbean and that a reintroduction of the B_PANDEMIC_ clade from the US generated the actual epidemic in the Caribbean region [22]. The authors recognize the existence of two major clusters in the Caribbean: cluster I (which grouped most of the sequences from Haiti, the Lesser Antilles, plus half of the Jamaican sequences) and cluster II (which included most of sequences from Cuba, Puerto Rico, and about half of the Jamaican sequences); but both lineages were associated to the B_PANDEMIC_ clade [22]. In our opinion, clusters I and II matched with the B_CAR_ and B_PANDEMIC_ clades here described, respectively. The clear distinction between pandemic and non-pandemic subtype B lineages was probably hampered in the previous study because the absence of reference subtype B sequences from US/Europe and the use of an unrooted phylogenetic tree.

The study of Holguin and Pagan (2013) proposes that the earliest subtype B Caribbean epidemics arose in the islands of Puerto Rico and Antigua around 1980 and that epidemics in Haiti, Dominican Republic and Jamaica only arose around the middle 1980s (95% HPD: 1980–1990). Our study and the study of Gilbert *et al* (2007), however, indicate that the subtype B epidemics in Hispaniola, Jamaica and Trinidad and Tobago probably arose before 1975. Of note, the mean evolutionary rate estimated for the HIV-1 *pol* gene in the previous study (3.6×10^−3^ subs/site/year) [22] was two times higher than the corresponding mean rate estimated here (1.7×10^−3^ subs/site/year). That rate was also higher than that usually estimated for the *pol* gene of HIV-1 subtype B (1.0–3.0×10^−3^ subs/site/year) [24], [38], [39], [40], [60], [61]. and other HIV-1 group M clades (1.0–2.5×10^−3^ subs/site/year) [24], [61], [62], [63], [64], [65], [66], [67], [68], [69], [70], [71], [72], [73]. That extremely fast calibration clock rate for the HIV-1 *pol* gene may have pushed T_MRCAs_ estimates of Caribbean epidemics toward misleading young ages.

The study of Holguin and Pagan (2013) also suggests that subtype B was mainly disseminated through the Caribbean following two routes: clade I would have jumped from Antigua to other Lesser Antilles, the Bahamas, Haiti and Jamaica; and clade II would have spread from Puerto Rico to Cuba, Jamaica, Haiti and Dominican Republic. Our study suggests a very different scenario in which the B_CAR_ clades (clade I) were disseminated from both Hispaniola and Trinidad and Tobago to the other Caribbean islands. We have not determined the most important hubs of dissemination of the B_PANDEMIC_ clade (clade II); but it is highly improbable to trace the origin of all B_PANDEMIC_ Caribbean sequences to Puerto Rico. The real scenario is probably more complex and other countries that maintain intensive migration/travel with the Caribbean like the US, England, Netherlands, France and Spain may have also acted as important hubs of dissemination of the B_PANDEMIC_ clade into the region.

Our demographic reconstruction suggests that B_CAR_ clades circulating in Hispaniola, Jamaica and Trinidad and Tobago experienced an initial phase of exponential growth followed by a more recent stabilization since the middle 1990s. This reconstructed demographic pattern fully agrees with the epidemiological profile of the Caribbean region, where the number of people living with HIV has remained relatively stable since the late 1990s [1], [6], and resemble the patterns previously described for subtype B epidemics in other American countries including Brazil [74] the US [75] and Panama [25]. Interestingly, the mean growth rates estimated for the B_CAR_ clades from Hispaniola, Jamaica and Trinidad and Tobago (0.35–0.45 year^−1^) were similar to those estimated for B_PANDEMIC_ clades mainly circulating among heterosexual populations from Panama (0.20–0.40 year^−1^) [25]; but lower than those estimated for B_PANDEMIC_ clades mainly transmitted among MSM populations from Cuba, Italy, Hong Kong and the United Kingdom (0.5–1.6 year^−1^) [24], [38], [39], [40]. This suggests that ecological factors, rather than viral lineage characteristics, are the major determinants of the HIV-1 subtype B epidemic growth rate across different countries.

In summary, this study demonstrates that non-pandemic HIV-1 subtype B viral strains have been widely disseminated through the Caribbean since the late 1960s and account for an important fraction (≥50%) of current HIV-1 infections in Haiti, Dominican Republic, Jamaica, the Bahamas and the Lesser Antilles. This study also indicates that Haiti, Dominican Republic and Trinidad and Tobago were probably the major hubs of dissemination of B_CAR_ in the region. Although this is the most comprehensive study of HIV-1 spread in the Caribbean performed to date, future studies would be improved by the use of more geographically balanced datasets as well as longer (ideally full-length) viral genomic sequences.

## Supporting Information

Table S1 **Distribution of HIV-1 subtype** ***pol*** **sequences from different countries within the B_PANDEMIC_ and the B_CAR_ clades.**
(PDF)

Table S2 **Number of sequence per location included in the complete and in the country “balanced” HIV-1 B_CAR_ datasets.**
(PDF)

Table S3 **Bayes factor (BF) rates of epidemiological links between locations for dispersal of non-pandemic B_CAR_ lineages in the Caribbean region.**
(PDF)

Table S4 **Mean estimated number of viral migrations between locations for dispersal of non-pandemic B_CAR_ lineages in the Caribbean region.**
(PDF)
