# Supplementary material for: Spatiotemporal Dynamics of Dissemination of Non-Pandemic HIV-1 Subtype B Clades in the Caribbean Region
Source: PLoS One. 2014 Aug 22;9(8):e106045. doi: 10.1371/journal.pone.0106045 (PMC4141835; doi:10.1371/journal.pone.0106045)
Supplement: Table S1 — Distribution of HIV-1 subtype pol sequences from different countries within the BPANDEMIC and the BCAR clades. (PDF) [file pone.0106045.s001.pdf]

Table S1. Distribution of HIV-1 subtype *pol* sequences from different countries within the $B_{PANDEMIC}$ and the B_{CAR} clades.

Region	Country	Total	$B_{PANDEMIC}$	B_{CAR}
Caribbean Greater Antilles	Cuba	319	316 (99.1%)	3 (0.9%)
	Puerto Rico	291	278 (95.5%)	13 (4.5%)
	Dominican Republic	168	44 (26.2%)	124 (73.8%)
	Jamaica	146	73 (50.0%)	73 (50.0%)
	Haiti	31	8 (25.8%)	23 (74.2%)
Caribbean Lesser Antilles	Martinique	452	268 (59.3%)	184 (40.7%)
	Guadeloupe	243	119 (49%)	124 (51%)
	US Virgin Islands	54	31 (57.4%)	23 (42.6%)
	Trinidad and Tobago	52	2 (3.9%)	50 (96.1%)
	Others*	39	8 (20.5%)	31 (79.5%)
Caribbean The Bahamas	Bahamas	11	6 (54.5%)	5 (45.5%)
North America	US	525	485 (89.7%)	54 (10.3%)
Europe	France	340	339 (99.7%)	1 (0.3%)

* Other Lesser Antilles: Antigua and Barbuda (n = 7), Barbados (n = 14), Dominica (n = 3), Grenada (n = 4), Montserrat (n = 1), Saint Lucia (n = 4) and Saint Vincent and the Grenadines (n = 6).
