# Supplementary material for: Spatiotemporal Dynamics of Dissemination of Non-Pandemic HIV-1 Subtype B Clades in the Caribbean Region
Source: PLoS One. 2014 Aug 22;9(8):e106045. doi: 10.1371/journal.pone.0106045 (PMC4141835; doi:10.1371/journal.pone.0106045)
Supplement: Table S2 — Number of sequence per location included in the complete and in the country “balanced” HIV-1 BCAR datasets. (PDF) [file pone.0106045.s002.pdf]

Table S2. Number of sequence per location included in the complete and in the country "balanced" HIV-1 B_{CAR} datasets.

Location	Complete	Subset 1	Subset 2	Subset 3	Subset 4	Subset 5
Hispaniola	136	25(A)	25(B)	25(C)	25(D)	25(E)
Jamaica	73	25(A)	25(B)	23(C)	25(A)	25(B)
Trinidad and Tobago	50	25(A)	25(B)	25(A)	25(B)	25(A)
Other*	20	20	20	20	20	20

We created five random non-overlapping subsets of sequences from Hispaniola (A to E), three from Jamaica (A to C) and two from Trinidad and Tobago (A and B) with up to 25 sequences per location. * Other Lesser Antilles: Antigua and Barbuda (*n* = 4), Dominica (*n* = 2), Grenada (*n* = 3), Montserrat (*n* = 1), Saint Lucia (*n* = 4) and Saint Vincent and the Grenadines (*n* = 6).
