# Supplementary material for: Spatiotemporal Dynamics of Dissemination of Non-Pandemic HIV-1 Subtype B Clades in the Caribbean Region
Source: PLoS One. 2014 Aug 22;9(8):e106045. doi: 10.1371/journal.pone.0106045 (PMC4141835; doi:10.1371/journal.pone.0106045)
Supplement: Table S3 — Bayes factor (BF) rates of epidemiological links between locations for dispersal of non-pandemic BCAR lineages in the Caribbean region. (PDF) [file pone.0106045.s003.pdf]

Table S3. Bayes factor (BF) rates of epidemiological links between locations for dispersal of non-pandemic B_{CAR} lineages in the Caribbean region.

**Locations**	**BF***
HIS-JM	35,401
TT-VC	2,079
HIS-DM	1,358
HIS-TT	284
HIS-AG	244
TT-GD	118
TT-MS	109
HIS-VC	91
TT-JM	53
HIS-SL	19
DRC-HIS	16
Others	<3

DRC: Democratic Republic of Congo; HIS: Hispaniola; JM: Jamaica; TT: Trinidad and Tobago; AG: Antigua and Barbuda; DM: Dominica; GD: Grenada; MS: Montserrat; SL: Saint Lucia; VC: Saint Vincent and the Grenadines. *BF > 100 indicates decisive support, 30 ≤ BF ≤ 100 indicates very strong support, 10 ≤ BF ≤ 30 indicates strong support, and 6 ≤ BF ≤ 10 indicates substantial support for migration between locations.
