# Supplementary material for: Spatiotemporal Dynamics of Dissemination of Non-Pandemic HIV-1 Subtype B Clades in the Caribbean Region
Source: PLoS One. 2014 Aug 22;9(8):e106045. doi: 10.1371/journal.pone.0106045 (PMC4141835; doi:10.1371/journal.pone.0106045)
Supplement: Table S4 — Mean estimated number of viral migrations between locations for dispersal of non-pandemic BCAR lineages in the Caribbean region. (PDF) [file pone.0106045.s004.pdf]

Table S4. Mean estimated number of viral migrations between locations for dispersal of non-pandemic B_{CAR} lineages in the Caribbean region.

**From**	**To**			
	HIS	JM	TT	LA
HIS	-	4.86	1.66	6.86
JM	0.69	-	0.23	2.04
TT	1.15	2.08	-	6.01
LA	0.09	0.07	0.05	-

HIS: Hispaniola; JM: Jamaica; TT: Trinidad and Tobago; LA: Antigua and Barbuda/Dominica/Grenada/Montserrat/Saint Lucia/Saint Vincent and the Grenadines.
